# Dietary *Lactobacillus plantarum* Supplementation Improves Growth and Modulates Hepatic and Immune-Related Responses in Tongue Sole (*Cynoglossus semilaevis*)

**DOI:** 10.3390/ani16071068

**Published:** 2026-04-01

**Authors:** Zipu Liu, Haien Zhang, Hongxiang Zhang, Weidong Li, Yangzhen Li, Yaotong Hao, Ran Guo

**Affiliations:** 1 Hebei Key Laboratory of Nutritional Regulation and Disease Control for Aquaculture, Ocean College, Hebei Agriculture University, Qinhuangdao 066003, China; 2 Tangshan Haidu Seafood Co., Ltd., Tangshan 063506, China; 3 Fishery Research Institute, Tangshan Academy of Agricultural Sciences, Tangshan 063001, China; 4 Yellow Sea Fisheries Research Institute, Chinese Academy of Fishery Sciences, Qingdao 266071, China

**Keywords:** hepatic function, antioxidant capacity, immune response, digestive enzyme activity

## Abstract

Lactic acid bacteria (LAB) are increasingly used as functional feed additives in aquaculture. This study evaluated the effects of dietary Lactobacillus plantarum supplementation on growth performance, hepatic biochemical status, lipid-related indices, antioxidant status, and immune-related responses in Chinese tongue sole (*Cynoglossus semilaevis*). A total of 1620 juveniles (initial body weight 8.11 ± 0.23 g) were randomly assigned to three dietary treatments for 10 weeks: a control diet (CON) and diets supplemented with 500 mg/kg (LAB1) or 1000 mg/kg (LAB2) LAB (three replicate tanks per treatment). Compared with CON, LAB supplementation improved growth performance, with LAB2 showing significantly higher final body weight, weight gain rate, and specific growth rate. LAB altered hepatic function-related indices and reduced hepatic lipid peroxidation, as indicated by lower malondialdehyde (MDA). Hepatic lipid-related indices were also modulated, with LAB2 showing reduced total cholesterol (TC), low-density lipoprotein cholesterol (LDL-C), triacylglycerol (TG), and total bile acid (TBA), together with increased high-density lipoprotein cholesterol (HDL-C). Serum immune indices showed non-linear responses, with C3, C4, and IgM increasing in LAB1 but decreasing in LAB2, whereas lysozyme showed an overall decreasing trend. qPCR analysis showed that LAB supplementation upregulated hepatic IGF-I and TGF-β1 expression, downregulated IL-8, TNF-α, and G6PD expression, and reduced FAS expression at the higher dose. Overall, dietary *L. plantarum* at 500–1000 mg/kg improved growth performance and was associated with changes in hepatic lipid-related indices, oxidative status, and immune-related responses in tongue sole. These results support further evaluation of *L. plantarum* as a functional feed additive in this species.

## Simple Summary

The use of probiotics in aquaculture is gaining attention as a sustainable way to improve fish growth and physiological condition while reducing reliance on antibiotics. In this study, we evaluated the effects of dietary *Lactobacillus plantarum* supplementation on juvenile Chinese tongue sole (*Cynoglossus semilaevis*) during a 10-week feeding trial. Fish fed *L. plantarum*-supplemented diets, particularly at 1000 mg/kg, showed better growth performance than those fed the control diet. Supplementation was also associated with changes in hepatic biochemical indices, reduced lipid peroxidation, altered lipid-related parameters, and modulation of immune-related indicators. In addition, the expression of genes associated with growth and immune regulation was significantly affected, suggesting that *L. plantarum* can influence both metabolic and inflammatory processes in tongue sole. Overall, these findings support further evaluation of *L. plantarum* as a functional feed additive for more sustainable tongue sole culture.

## 1. Introduction

Fish reared under intensive aquaculture systems are routinely exposed to multiple stressors, including high stocking density, fluctuating water quality, handling, and pathogen pressure, that can disrupt physiological homeostasis and increase the risk of infectious disease outbreaks, thereby causing substantial economic losses [1,2]. To control bacterial diseases under intensive production conditions, antibiotics have historically been widely used [3,4]. However, extensive antibiotic use has raised increasing concerns, including the accumulation of antibiotic residues in aquatic products and the surrounding environment [5] and the emergence and spread of antibiotic-resistant bacteria [6]. Accordingly, regulatory authorities in many countries have tightened restrictions or prohibited the use of certain antibiotics in aquaculture [7,8,9,10]. These constraints have stimulated the development of environmentally friendly disease-management strategies in aquaculture [11]. In this context, functional feed additives, including probiotics, antimicrobial peptides, traditional Chinese herbal medicines, and plant-derived extracts, have attracted growing interest as alternatives or complements to antibiotics, with the aim of improving fish growth, health status, and resilience to stress and infection [11,12,13].

Probiotics are generally defined as live microorganisms that, when administered in adequate amounts, confer a health benefit on the host [14]. In fish, dietary probiotics are most commonly used to support intestinal function by shaping gut microbial communities, enhancing barrier integrity, and improving nutrient utilization [15,16]. A growing body of evidence indicates that probiotic supplementation can improve survival, growth performance, and feed efficiency, while also enhancing antioxidant capacity, immune responsiveness, and resistance to bacterial pathogens [17]. In aquaculture practice, probiotics can be delivered through feed or applied directly to the culture water, depending on the product and production system [18].

Among probiotic candidates, lactic acid bacteria (LAB) are widely used because of their favorable safety profile, ecological ubiquity, and ability to produce organic acids and other antimicrobial compounds. LAB are typically Gram-positive, non-spore-forming bacteria that are catalase-negative or weakly catalase-positive and include diverse morphotypes such as rods and cocci [19,20]. In aquaculture, LAB have been reported to promote growth and feed utilization, improve antioxidant status, stimulate innate immune responses, and reduce susceptibility to disease, partly via competitive exclusion of pathogens and regulation of host-associated microbial communities [21,22,23,24,25]. Common LAB used in aquaculture include *Lactococcus lactis* [26,27,28], *Lactobacillus plantarum* [27,29], *Pediococcus acidilactici* [25], and *Lactobacillus rhamnosus* [26,28]. Among these, *L. plantarum* was selected for the present study because it is one of the most commonly applied LAB probiotics in aquaculture and has shown beneficial effects on growth, antioxidant status, immune-related responses, and disease resistance in fish [27,29].

Chinese tongue sole (*Cynoglossus semilaevis*) is an economically important marine flatfish and a high-value cultured species in China [30,31]. Although LAB supplementation has shown favorable effects on growth, antioxidant capacity, immune indices, disease resistance, and microbial homeostasis in multiple aquaculture species, the responses of flatfish, and particularly tongue sole, remain insufficiently characterized. Evaluating hepatic endpoints is biologically relevant because the liver is a central organ for intermediary metabolism, lipid homeostasis, detoxification, and systemic immune regulation. This focus is especially relevant in flatfish aquaculture, where intensive culture conditions and formulated feeds can place a substantial metabolic burden on the liver, making hepatic biochemical responses sensitive indicators of nutritional intervention. In addition, the gut-liver axis may provide one possible framework for understanding probiotic effects on hepatic physiology, because microbial products and metabolites may influence hepatic oxidative status, inflammatory signaling, and lipid metabolism through the portal circulation.

Nevertheless, most probiotic studies in fish have primarily focused on intestinal outcomes (e.g., gut microbiota composition, digestive function, and mucosal immunity), whereas investigations specifically targeting liver functions are comparatively scarce, especially in tongue sole. Because no intestinal microbiota or probiotic colonization data were measured in the present study, gut-liver interactions are discussed only as a possible explanatory context rather than a demonstrated mechanism. Therefore, the present study conducted a 10-week feeding trial to evaluate the effects of dietary LAB supplementation on tongue sole, with a particular focus on hepatic physiological and biochemical indices and the expression of immune-related genes, alongside growth performance. We hypothesized that dietary *L. plantarum* supplementation would improve growth performance and modulate hepatic biochemical, oxidative, and immune-related responses in tongue sole in a dose-dependent manner.

## 2. Materials and Methods

### 2.1. Experimental Diets Preparation

Three isonitrogenous and isolipidic diets were formulated to contain approximately 53% crude protein and 8% crude lipid (approximately 3% crude fiber and 16% crude ash). The basal diet formulation used in this study was the same as that previously described by Li et al. [32]. Dietary *L. plantarum* was supplemented at 0 (control, CON), 500 (LAB1), or 1000 mg/kg diet (LAB2), and these two supplementation levels were selected according to the manufacturer’s recommendation. The probiotic was provided as a commercial live-cell powder preparation sup-plied by Santong Bioengineering (Weifang) Co., Ltd. (Weifang, China), with a stated potency of 6 × 10^10^ CFU/g. Strain-level information beyond the commercial product label was not provided by the manufacturer. To prepare the experimental diets, the required amount of probiotic powder was first premixed with a small portion of the basal diet to improve homogeneity and then incorporated into the remaining feed before pelleting. Purified water (50 mL/kg diet) was added, and the mixture was thoroughly blended. Pellets were dried in a ventilated oven at 35 °C for 24 h and stored at room temperature until use. According to the manufacturer, the resulting viable counts were approximately 3.1 × 10^7^ CFU/g in the LAB1 diet and 6.1 × 10^7^ CFU/g in the LAB2 diet, whereas no colonies were detected in the control diet. However, probiotic viability during storage and throughout the feeding trial was not independently monitored in the present study; therefore, the actual viable dose consumed by the fish may have differed from the nominal supplemented dose.

### 2.2. Fish and Feeding Trial

Juvenile Chinese tongue soles were obtained from Tangshan Haidu Aquatic Products Co., Ltd. (Tangshan, China). Fish were acclimated for two weeks and fed the control diet before the trial. After acclimation, fish were randomly distributed into nine 2 m^3^ tanks (three replicate tanks per dietary treatment). Each tank contained 180 fish. Fish were hand-fed twice daily (08:00 and 20:00) to apparent satiation, with an estimated daily ration of 1–2% of wet body weight, for 10 weeks. Uneaten feed and feces were removed daily. Seawater was renewed at approximately 500% tank volume per day. During the feeding trial, water temperature was maintained at 22 ± 0.5 °C, salinity was approximately 28‰, dissolved oxygen remained above 5 mg/L, and pH ranged from 7.4 to 7.8.

### 2.3. Growth Performance and Indices

At the beginning and end of the 10-week feeding period, fish in each tank were fasted for 24 h prior to measurement. Thirty fish were randomly sampled from each tank to measure individual body weight. Initial body weight (BW_i_), final body weight (BW_f_), and the number of surviving fish were recorded for each tank. Growth performance indices were calculated as follows:

$$weight\;gain\;rate\left(WGR,\%\right)=\frac{{BW}_{f}-{BW}_{i}}{{BW}_{i}}\times 100;$$

$$specific\;growth\;rate\;(SGR,\%/day),={(e}^{\frac{{log}_{e}\left({BW}_{f}\right)-{log}_{e}\left({BW}_{i}\right)}{days}}-1)\times 100;$$

$$hepatosomatic\;index\left(HSI,\%\right)=\frac{liver\;weight}{body\;weight}\times 100;$$

$$survival\;rate\left(SR,\%\right)=\frac{N1}{N0}\times 100\%,$$

where N0 and N1 represent the initial and final number of fish per tank, respectively.

### 2.4. Sample Collection

At the end of the trial, fish were fasted for 24 h. Ten fish were randomly selected from each tank and anesthetized with MS-222 (20 mg/L) for sampling. Fish were weighed, and liver samples were excised and weighed for HSI calculation. For biochemical and molecular analyses, liver tissue and blood were collected from three fish per tank. Samples from individual fish were processed separately, and the mean value of the three fish within each tank was used as the tank-level value for statistical analysis. Liver samples were divided into aliquots, snap-frozen in liquid nitrogen, and stored at −80 °C until analysis. Blood samples were collected from the caudal vein (~0.2 mL per fish) using sterile syringes, allowed to clot at 4 °C, and centrifuged at 3000× *g* for 10 min to obtain serum. Serum was aliquoted and stored at −80 °C.

### 2.5. Physiological and Biochemical Analyses

Frozen liver and serum samples were thawed on ice prior to analysis. Commercial diagnostic kits (Nanjing Jiancheng Bioengineering Institute, Nanjing, China) were used to measure hepatic and serum biochemical indices according to the manufacturer’s protocols. The measured parameters included indices related to hepatic lipid metabolism, antioxidant capacity, liver function, digestive enzyme activities, and serum immune-related biomarkers. The complete list of parameters and corresponding kits is provided in Table 1. Because the commercial kits use assay-specific definitions, the activity units reported for protease, lipase, and amylase are not identical.

### 2.6. RNA Isolation and Quantitative Real-Time PCR (qRT-PCR) Analysis

Hepatic mRNA expression of three growth/metabolism-related genes (IGF-I, FAS, and G6PD) and three immune-related genes (IL-8, TGF-β1, and TNF-α) was determined by qRT-PCR. Total RNA was extracted from liver tissue using TRIzol reagent (Takara, Kyoto, Japan) following the manufacturer’s instructions. RNA integrity was assessed by 1.2% agarose gel electrophoresis, and concentration/purity were evaluated using a Nanodrop 2000 spectrophotometer (Thermo Fisher Scientific, Waltham, MA, USA). cDNA was synthesized using a Servicebio^®^ RT reagent kit with gDNA Eraser (Servicebio, Wuhan, China). Gene-specific primers were designed using Premier 5.0 and are listed in Table 2. qRT-PCR was performed on a QuantStudio™ 5 system (Thermo Scientific, Waltham, MA, USA) with three technical triplicates. Reaction conditions and cycling programs followed [33]. Relative gene expression was calculated using the 2^−ΔΔCt^ method [34], with β-actin used as the reference gene and the control group (CON) as the calibrator.

### 2.7. Statistical Analysis

All growth performance, biochemical indices, and gene expression data were analyzed using tank as the experimental unit (three tanks per treatment). Data are presented as mean ± standard error (SE). Prior to analysis, percentage data were arcsine square-root transformed when appropriate. Normality and homogeneity of variances were evaluated using the Shapiro-Wilk test and Levene’s test, respectively, before one-way analysis of variance (ANOVA) was performed. Differences among dietary treatments were further compared using Tukey’s multiple comparison test. Statistical analyses were performed using R (version 4.1.0). Differences were considered significant at *p* < 0.05.

## 3. Results

### 3.1. Growth, Hepatosomatic Index and Survival

Growth performance, HSI, and SR are summarized in Table 3. BWi did not differ among treatments at the start of the trial (*p* > 0.05). After 10 weeks, dietary LAB supplementation increased BW_f_, WGR, and SGR. Fish in the LAB2 group showed significantly higher BW_f_, WGR, and SGR compared with the control group (*p* < 0.05), whereas LAB1 values were intermediate and did not differ significantly from either CON or LAB2 (*p* > 0.05). No significant differences were detected in HSI or SR among groups (*p* > 0.05).

### 3.2. Hepatic Function-Related Indices

Hepatic function-related indices are shown in Table 4. UN was significantly higher in LAB1 than in CON and LAB2 (*p* < 0.05). ALB was significantly reduced in LAB2 compared with CON and LAB1 (*p* < 0.05). ALT decreased with increasing LAB supplementation, with LAB2 showing the lowest ALT activity (*p* < 0.05). AST displayed a non-linear pattern, it increased in LAB1 relative to CON, then decreased to the lowest level in LAB2 (*p* < 0.05).

### 3.3. Hepatic Lipid Metabolism

Hepatic lipid metabolism parameters are presented in Table 5. TBA was significantly lower in LAB2 than in CON and LAB1 (*p* < 0.05). TC and LDL-C decreased progressively with increasing LAB supplementation, with the lowest values observed in LAB2 (*p* < 0.05). TG levels differed among treatments, showing a higher value in LAB1 compared with CON, but a marked reduction in LAB2 (*p* < 0.05). HDL-C increased in LAB-supplemented fish, reaching the highest level in LAB1, and remaining above the control in LAB2 (*p* < 0.05).

### 3.4. Hepatic Digestive Enzyme Activities

Activities of hepatic protease, lipase, and amylase are shown in Table 6. Compared with the control group, LAB supplementation significantly increased the activities of all three enzymes (*p* < 0.05). Moreover, LAB2 exhibited significantly higher protease, lipase, and amylase activities than LAB1 (*p* < 0.05), indicating a dose-dependent increase across the tested supplementation levels.

### 3.5. Hepatic Antioxidant Status

Hepatic antioxidant indices are summarized in Table 7. SOD activity decreased stepwise from CON to LAB1 and LAB2 (*p* < 0.05). CAT did not differ between CON and LAB1 (*p* > 0.05) but was significantly lower in LAB2 (*p* < 0.05). TAOC increased in LAB1 compared with CON, but returned to a level similar to CON in LAB2 (*p* < 0.05 among treatments). GSH-PX activity and MDA content decreased with increasing LAB supplementation, with the lowest values in LAB2 (*p* < 0.05).

### 3.6. Serum Immune-Related Indices

**Figure 1:**
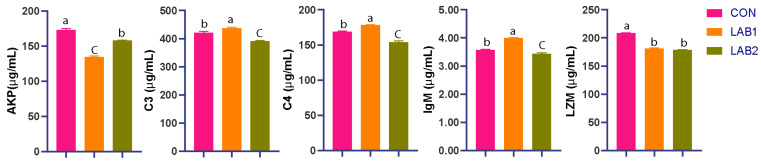
The effects of dietary lactic acid bacteria supplementation on the activity of immune enzymes in the blood of tongue sole. Values are the mean ± SE (three tanks per treatment). Different letters indicate significant differences among treatments (*p* < 0.05). CON: 0 mg/kg; LAB1: 500 mg/kg; LAB2: 1000 mg/kg LAB.

Serum immune-related indices are shown in Figure 1. AKP activity was significantly reduced by LAB supplementation, with the lowest value observed in LAB1 (*p* < 0.05). C3, C4, and IgM showed non-linear responses, increasing in LAB1 but decreasing in LAB2 (*p* < 0.05). LZM tended to decrease in LAB-supplemented groups, although the difference between LAB1 and LAB2 was not significant (*p* > 0.05).

### 3.7. Hepatic Expression of Growth- and Immune-Related Genes

**Figure 2:**
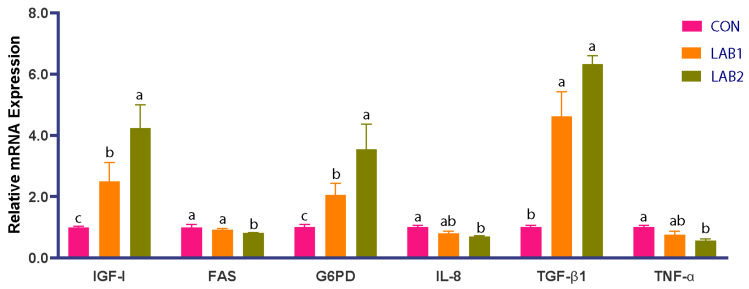
Relative expression of growth- and immune-related genes in liver of tongue sole after 10 weeks of feeding LAB-supplemented diets. Values are the mean ± SE (three tanks per treatment). Different letters indicate significant differences among treatments (*p* < 0.05). CON: 0 mg/kg; LAB1: 500 mg/kg; LAB2: 1000 mg/kg LAB.

Relative hepatic gene expression is shown in Figure 2. LAB supplementation significantly upregulated IGF-I expression in both LAB1 and LAB2 compared with CON (*p* < 0.05). In contrast, G6PD expression was significantly downregulated in both LAB groups (*p* < 0.05). FAS expression showed a dose-dependent suppression, with a nonsignificant decrease in LAB1 and a significant reduction in LAB2 relative to CON (*p* < 0.05). For immune-related genes, LAB supplementation decreased the expression of the pro-inflammatory cytokines IL-8 and TNF-α in a dose-dependent manner (*p* < 0.05), whereas the anti-inflammatory cytokine TGF-β1 was significantly upregulated in LAB-supplemented groups (*p* < 0.05).

## 4. Discussion

### 4.1. Beneficial Effects of LAB Supplementation on Growth Performance and Digestive Capacity

LAB strains have been widely applied as probiotics in aquaculture and are frequently reported to improve growth performance, particularly during early life stages. These benefits are generally attributed to enhanced nutrient utilization, modulation of host-associated microbial communities, improved intestinal function, and stimulation of digestive and metabolic processes, rather than direct nutritional contributions of the probiotic itself [35,36]. In the present study, dietary LAB supplementation improved growth performance of tongue sole, with LAB2 showing significantly higher BW_f_, WGR, and SGR than the control group. This result is consistent with previous reports in other teleost, including red sea bream (*Pagrus major*) [37], snakehead (*Channa argus*) [38], and striped snakehead (*Channa striata*) [39].

Consistent with the observed growth enhancement, hepatic expression of the growth-regulating gene IGF-1 was significantly upregulated in LAB-supplemented fish, suggesting that LAB inclusion was associated with a physiological state supporting anabolic growth. Similar growth-promoting effects have been reported for other LAB strains; for example, supplementation of *Lactococcus lactis* (1 × 10^6^ CFU/g) for 56 days significantly improved BW_f_, WGR, and SGR in red sea bream [37]. In Nile tilapia (*Oreochromis niloticus*), dietary inclusion of *Lactobacillus paracasei* also improved growth performance and reduced feed conversion ratio [40], supporting the view that LAB can improve feed utilization in diverse fish species.

We further observed that LAB supplementation significantly increased the activities of protease, lipase, and amylase measured in liver tissue. Although digestive enzymes are more commonly assessed in intestine and pancreas, changes in hepatic enzyme activities may reflect broader shifts in nutrient handling and metabolic capacity under LAB supplementation. A related phenomenon has been reported in grouper (*Epinephelus coioides*), where dietary probiotics (including *Enterococcus faecalis* and *Lactobacillus*) increased liver trypsin activity [41]. Notably, probiotic effects can be strain- and host-dependent, and not all LAB strains necessarily enhance enzyme activities; thus, additional studies are required to clarify strain specificity and tissue specificity of these responses.

### 4.2. Beneficial Effects of LAB Supplementation on Hepatic Function-Related Indices and Antioxidant Status

ALT and AST are aminotransferases involved in amino acid transamination and intermediary carbon and nitrogen metabolism. When measured in serum they are often used as indicators of hepatocellular injury, but in the present study they were quantified in liver homogenates and are therefore more appropriately interpreted as hepatic enzyme activities rather than serum clinical markers. The progressive decrease in hepatic ALT activity with increasing LAB inclusion may indicate reduced transamination demand or altered amino acid utilization in liver tissue under LAB supplementation. In contrast, hepatic AST increased at LAB1 and then decreased markedly at LAB2, suggesting a dose-dependent adjustment of hepatic metabolic flux rather than a simple linear response. Because these measurements were not accompanied by serum biochemistry or liver histology, they should not be taken as direct evidence of reduced liver injury. Instead, they indicate that LAB supplementation was associated with altered hepatic metabolic status.

Oxidative stress can impair cellular function, compromise immunity, and increase susceptibility to disease in cultured fish. MDA is commonly used as an index of lipid peroxidation, and elevated MDA reflects oxidative damage [42]. In the present study, hepatic MDA decreased significantly with LAB supplementation, with the lowest level in LAB2, indicating lower lipid peroxidation. Antioxidant enzymes including SOD, CAT, and GSH-PX are important components of the endogenous defense system [43]. However, their activities declined as LAB dose increased, whereas T-AOC increased in LAB1 but returned to approximately the control level in LAB2. Taken together, these results suggest that the hepatic antioxidant response under LAB supplementation was complex. Although the reduction in MDA is consistent with lower oxidative damage, the concurrent decrease in major antioxidant enzymes should not be interpreted as unequivocally beneficial, because it may reflect either reduced oxidative demand or altered antioxidant defense capacity. Additional oxidative stress markers and functional assays will be needed to clarify this mechanism.

At the transcriptional level, G6PD was significantly downregulated in LAB-supplemented fish. As G6PD is a key enzyme in the pentose phosphate pathway that supplies NADPH for redox regulation and biosynthesis, reduced expression could be consistent with a reduced oxidative challenge and lower demand for NADPH-dependent antioxidant regeneration. G6PD has also been linked to immune-related oxidative metabolism in leukocytes [44], suggesting potential metabolic-immune interactions. Overall, these findings indicate that LAB supplementation was associated with changes in hepatic oxidative status and antioxidant-related metabolism, with LAB2 showing the lowest MDA content.

### 4.3. Beneficial Effects of LAB Supplementation on Lipid Metabolism

The liver is a central organ for lipid metabolism in fish, coordinating lipid synthesis, catabolism, and transport [45]. In the present study, LAB supplementation significantly influenced hepatic lipid-related indices. LAB2 exhibited markedly reduced hepatic TC, LDL-C, and TG relative to the control, while HDL-C increased in LAB-supplemented fish (highest in LAB1 and remaining above the control in LAB2). These changes suggest that LAB supplementation was associated with altered hepatic lipid homeostasis and potentially reduced lipid accumulation.

LAB strains have been proposed to influence cholesterol metabolism through multiple mechanisms, including assimilation of cholesterol, binding/co-precipitation with bile salts, and enzymatic conversion (e.g., bile salt hydrolase-related pathways). In our study, TBA was significantly reduced in LAB2. Because bile acids are terminal metabolites of cholesterol catabolism and participate in enterohepatic circulation [46], reduced hepatic TBA may reflect modulation of bile acid synthesis/transport/recirculation rather than a simple “increase” or “decrease” in cholesterol breakdown. Similar alterations in bile-acid-related indices under probiotic interventions have been reported [47]. In addition, LDL-C and HDL-C are often considered informative indicators of lipid transport status and can be associated with metabolic disturbances when abnormal [48,49]. The overall pattern observed here, decreased LDL-C and increased HDL-C, may indicate a shift toward a more favorable lipid profile under LAB supplementation.

At the gene level, hepatic FAS expression was suppressed, particularly at the higher LAB dose. Since FAS is a key lipogenic enzyme, its downregulation is consistent with reduced lipogenesis and with the lower hepatic TC and TG observed in LAB2. Mechanistically, these effects may be linked to changes in host metabolism and possibly to gut-liver interactions, but this remains speculative because gut microbiota, probiotic colonization, and bile-acid pathway genes were not measured in the present study.

### 4.4. Beneficial Effects of LAB Inclusion on Serum Immune Indices and Hepatic Cytokine Expression

The fish immune system is a critical defense mechanism against pathogens and environmental challenges [50]. IgM and complement components (C3 and C4) are widely used indicators of innate and humoral immune status [51,52]. In the present study, serum IgM, C3, and C4 showed a non-linear pattern, increasing in LAB1 and decreasing in LAB2. This pattern indicates that circulating humoral immune factors responded differently at the two inclusion levels. The higher values in LAB1 may reflect stimulation of selected serum immune components, whereas the decline in LAB2 may reflect attenuation or regulatory feedback rather than clear immune enhancement. Because no pathogen challenge or functional immune assay was performed, the lower serum values at the higher dose should not be interpreted either as definitive immunosuppression or as improved immune status [53,54]. A cautious interpretation is that different LAB inclusion levels differentially modulated circulating immune-related indices.

Cytokines are key regulators of immune and inflammatory processes and include interleukins, TNFs, interferons, transforming growth factors, and chemokines [19]. TNF-α and IL-8 are important pro-inflammatory mediators involved in innate immune responses and tissue repair [55], whereas TGF-β1 is a major immunoregulatory cytokine that can suppress excessive inflammation [56]. In contrast to the non-linear serum responses, hepatic cytokine expression showed a more consistent dose-related pattern in the present study: dietary LAB supplementation downregulated hepatic IL-8 and TNF-α and upregulated TGF-β1. This cytokine pattern is consistent with a shift toward a less pro-inflammatory hepatic signaling profile under LAB supplementation. Similar probiotic-induced modulation of cytokine genes has been reported; for example, LAB supplementation altered both pro- and anti-inflammatory cytokine expression in immune tissues of *Micropterus salmoides* [57].

Taken together, the serum immune indices and hepatic cytokine expression suggest that LAB supplementation modulated immune-related responses in tongue sole, but the patterns differed between circulating serum factors and hepatic cytokine transcription. However, whether these changes translate into improved disease resistance under farming conditions requires further verification through pathogen challenge trials and/or functional immune assays.

Several limitations of the present study should be acknowledged. First, although approximate CFU/g values for the supplemented diets were provided by the manufacturer, probiotic viability in the finished diets was not independently verified during storage or throughout the feeding trial; therefore, the actual viable dose consumed by the fish remains uncertain. Second, neither gut microbiota composition nor probiotic colonization was assessed, so any gut-liver interactions discussed here should be considered hypothetical rather than demonstrated. Third, no liver histology was performed, and thus the hepatic effects reported here are supported by biochemical and gene-expression data rather than direct morphological evidence. Finally, no pathogen challenge or functional immune assay was conducted, so the observed immune-related changes cannot be interpreted as confirmed enhancement of disease resistance.

## 5. Conclusions

Dietary supplementation with Lactobacillus plantarum (500–1000 mg/kg) improved growth performance of juvenile tongue sole during the 10-week feeding trial, with the 1000 mg/kg diet producing the highest BW_f_, WGR, and SGR. LAB supplementation was also associated with modulation of hepatic function- and lipid-related indices, reduced hepatic lipid peroxidation, non-linear changes in serum immune-related factors, and altered hepatic expression of IGF-I, FAS, G6PD, IL-8, TGF-β1, and TNF-α. These results suggest that dietary *L. plantarum* can influence growth and multiple physiological indicators in tongue sole; however, the hepatic effects reported here are based on biochemical and molecular measurements rather than histological validation. Future studies should verify probiotic viability in finished diets, evaluate gut microbiota responses and liver histology, and test whether these changes translate into enhanced disease resistance under pathogen challenge.

## Figures and Tables

**Table 1:** The assayed hepatic and serum parameters in this study.

Item	Index (Activity or Content)	Version
Digestive enzyme	Protease	A080-2-2
	Lipase	A054-1-1
	Amylase	C016-2-1
Lipid metabolism	Total bile acid (TBA)	E003-2-1
	Triacylglycerol (TG)	A110-1-1
	Total cholesterol (TC)	A111-1-1
	High-density lipoprotein cholesterol (HDL-C)	A112-1-1
	Low-density lipoprotein cholesterol (LDL-C)	A113-1-1
Antioxidant index	Superoxide dismutase (SOD)	A001-3-2
	Catalase (CAT)	A007-2-1
	Total antioxidant capacity (TAOC)	A015-2-1
	Glutathione peroxidase (GPX)	H545-1-2
	Malondialdehyde (MDA)	A003-3-1
Hepatic function	Urea nitrogen (UN)	C013-2-1
	Albumin (ALB)	A028-2-1
	Alanine transaminase (ALT)	C009-2-1
	Aspartate aminotransferase (AST)	C010-2-1
Serum immune	Complement 3 (C3)	E032-1-1
	Complement 4 (C4)	E033-1-1
	Lysozyme assay (LZM)	A050-1-1
	Immunoglobulin M (IgM)	E025-1-1
	Alkaline phosphatase (AKP)	A059-2-2

**Table 2:** Primers used in quantitative real-time PCR (qRT-PCR).

Gene	Forward Primer (5′-3′)	Reverse Primer (5′-3′)	Product Length	Accession No.
β-actin	CCAACAGGGAAAAGATGACC	TTCTCCTTGATGTCACGCAC	304	NM_001308179
IGF-I	TGGGATGTTCTCAAGAGTGCG	GGTTTGCTGAAATAAAAGCCTCTC	182	NM_001294198.1
FAS	GCTGCTCAAGCCAAACACCT	CCCTGCTCTTTGTAGCCGTCT	210	XM_008314383.3
G6PD	TGCTCCCAGACAACACCTACTTT	ACAATCACTCTGTTCCAGCCTCT	328	XM_008318150.3
IL-8	CTGAAGGAATGAGCCTGAGAAGC	TCACTTTCTTCACCCAAGGAGC	207	XM_025058206.1
TGF-β1	GATGTGACTGAGACCCTGCAGAAC	TCCCAAAAGAGACCCCAGAGAT	142	XM_008308810.3
TNF-α	TGTGAGAGCGGCCATTCATT	GGAACGACACCTGGCTGTAA	173	XM_008331806.3

β-actin: Beta-actin; IGF-I: Insulin-like Growth Factor I; FAS: Fatty Acid Synthase; G6PD: Glucose-6-Phosphate Dehydrogenase; IL-8: Interleukin-8; TGF-β1: Transforming Growth Factor Beta 1; TNF-α: Tumor Necrosis Factor Alpha.

**Table 3:** Growth performance, hepatosomatic index (HSI), and survival rate (SR) of tongue sole fed diets supplemented with LAB for 10 weeks. Values are the mean ± SE (three tanks per treatment). Different superscript letters within a row indicate significant differences (*p* < 0.05). CON: 0 mg/kg; LAB1: 500 mg/kg; LAB2: 1000 mg/kg LAB.

Group	CON	LAB1	LAB2
BW_i_ (g)	8.00 ± 0.12 ^a^	8.22 ± 0.12 ^a^	8.19 ± 0.09 ^a^
BW_f_ (g)	70.58 ± 2.86 ^b^	79.99 ± 2.47 ^ab^	87.15 ± 2.53 ^a^
WGR (%)	781.71 ± 59.64 ^b^	882.50 ± 73.37 ^ab^	962.61 ± 58.62 ^a^
SGR (%/day)	3.88 ± 0.12 ^b^	4.08 ± 0.13 ^ab^	4.22 ± 0.10 ^a^
HSI (%)	1.08 ± 0.07 ^a^	1.11 ± 0.07 ^a^	1.14 ± 0.11 ^a^
SR (%)	86.88 ± 1.40 ^a^	87.76 ± 0.48 ^a^	89.76 ± 0.81 ^a^

**Table 4:** The impact of dietary LAB supplementation on liver function indicators of tongue sole. Values are the mean ± SE (three tanks per treatment). Different superscript letters within a row indicate significant differences (*p* < 0.05). CON: 0 mg/kg; LAB1: 500 mg/kg; LAB2: 1000 mg/kg LAB.

Group	CON	LAB1	LAB2
UN (pmol/mg prot)	1.26 ± 0.02 ^c^	2.00 ± 0.01 ^a^	1.55 ± 0.01 ^b^
ALB (μg/mg prot)	1.39 ± 0.04 ^a^	1.33 ± 0.01 ^a^	0.75 ± 0.02 ^b^
ALT (U/g prot)	196.02 ± 5.48 ^a^	149.98 ± 0.37 ^b^	102.12 ± 3.29 ^c^
AST (U/g prot)	459.07 ± 6.57 ^b^	675.79 ± 10.98 ^a^	359.73 ± 7.01 ^c^

**Table 5:** Effects of dietary LAB on lipid metabolism in liver in tongue sole. Values are the mean ± SE (three tanks per treatment). Different superscript letters within a row indicate significant differences (*p* < 0.05). CON: 0 mg/kg; LAB1: 500 mg/kg; LAB2: 1000 mg/kg LAB.

Group	CON	LAB1	LAB2
TBA (μmol/g prot)	121.98 ± 1.83 ^a^	130.95 ± 2.10 ^a^	96.57 ± 0.37 ^b^
TC (μmol/mg prot)	4.61 ± 0.08 ^a^	3.67 ± 0.05 ^b^	2.22 ± 0.06 ^c^
TG (μmol/mg prot)	1.12 ± 0.03 ^b^	1.34 ± 0.03 ^a^	0.66 ± 0.01 ^c^
LDL-C (μmol/mg prot)	1.84 ± 0.05 ^a^	1.54 ± 0.04 ^b^	1.09 ± 0.01 ^c^
HDL-C (μmol/mg prot)	0.89 ± 0.03 ^c^	1.29 ± 0.03 ^a^	1.04 ± 0.01 ^b^

**Table 6:** Effects of dietary LAB on the activity levels of protease, lipase and amylase in liver in tongue sole. Values are the mean ± SE (three tanks per treatment). Different superscript letters within a row indicate significant differences (*p* < 0.05). CON: 0 mg/kg; LAB1: 500 mg/kg; LAB2: 1000 mg/kg LAB.

Group	CON	LAB1	LAB2
Protease (U/mg prot)	3.75 ± 0.09 ^c^	5.80 ± 0.05 ^b^	6.48 ± 0.04 ^a^
Lipase (mU/g prot)	91.78 ± 13.18 ^c^	138.01 ± 0.19 ^b^	175.18 ± 2.61 ^a^
Amylase (U/g prot)	7.63 ± 0.09 ^c^	10.78 ± 0.13 ^b^	15.29 ± 0.23 ^a^

**Table 7:** Effects of dietary LAB on liver antioxidant capacity in tongue sole. Values are the mean ± SE (three tanks per treatment). Different superscript letters within a row indicate significant differences (*p* < 0.05). CON: 0 mg/kg; LAB1: 500 mg/kg; LAB2: 1000 mg/kg LAB.

Group	CON	LAB1	LAB2
SOD (U/μg prot)	0.51 ± 0.01 ^a^	0.42 ± 0.00 ^b^	0.33 ± 0.01 ^c^
CAT (U/mg prot)	101.09 ± 1.66 ^a^	103.63 ± 0.59 ^a^	65.26 ± 1.10 ^b^
TAOC (ng/g prot)	222.97 ± 3.02 ^b^	303.88 ± 6.22 ^a^	220.15 ± 1.13 ^b^
GSH-PX (mU/g prot)	332.46 ± 5.68 ^a^	263.02 ± 0.88 ^b^	177.47 ± 5.54 ^c^
MDA (pmol/μg prot)	49.70 ± 1.41 ^a^	46.13 ± 0.88 ^b^	36.25 ± 0.40 ^c^

## Data Availability

The original contributions presented in the study are included in the article. Further inquiries can be directed at the corresponding author.

## Abbreviations

The following abbreviations are used in this manuscript:

BW_i_	Initial body weight
BW_f_	Final body weight
WGR	Weight gain rate
SGR	Specific growth rate
HSI	Hepatosomatic index
SR	Survival rate
TBA	Total bile acid
TG	Triacylglycerol
TC	Total cholesterol
HDL-C	High-density lipoprotein cholesterol
LDL-C	Low-density lipoprotein cholesterol
SOD	Superoxide dismutase
CAT	Catalase
TAOC	Total antioxidant capacity
GPX	Glutathione peroxidase
MDA	Malondialdehyde
UN	Urea nitrogen
ALB	Albumin
ALT	Alanine transaminase
AST	Aspartate aminotransferase
C3	Complement 3
C4	Complement 4
LZM	Lysozyme assay
IgM	Immunoglobulin M
AKP	Alkaline phosphatase
IGF-I	Insulin-like Growth Factor
FAS	Fatty Acid Synthase
G6PD	Glucose-6-Phosphate Dehydrogenase
IL-8	Interleukin-8
TGF-β1	Transforming Growth Factor Beta 1
TNF-α	Tumor Necrosis Factor Alpha
β-actin	Beta-actin

## References

[1] Mukherjee A., Chandra G., Ghosh K. (2019). Single or conjoint application of autochthonous Bacillus strains as potential probiotics: Effects on growth, feed utilization, immunity and disease resistance in Rohu, *Labeo rohita* (Hamilton). Aquaculture.

[2] Lafferty K.D., Harvell C.D., Conrad J.M., Friedman C.S., Kent M.L., Kuris A.M., Powell E.N., Rondeau D., Saksida S.M. (2015). Infectious diseases affect marine fisheries and aquaculture economics. Annu. Rev. Mar. Sci..

[3] Chen J., Sun R.X., Pan C.G., Sun Y., Mai B.X., Li Q.X. (2020). Antibiotics and food safety in aquaculture. J. Agric. Food Chem..

[4] Limbu S.M., Chen L.Q., Zhang M.L., Du Z.Y. (2021). A global analysis on the systemic effects of antibiotics in cultured fish and their potential human health risk: A review. Rev. Aquac..

[5] Song C., Zhang C., Kamira B., Qiu L., Fan L., Wu W., Meng S., Hu G., Chen J. (2017). Occurrence and human dietary assessment of fluoroquinolones antibiotics in cultured fish around Tai Lake, China. Environ. Toxicol. Chem..

[6] Squadrone S. (2020). Water environments: Metal-tolerant and antibiotic-resistant bacteria. Environ. Monit. Assess..

[7] Assefa A., Abunna F. (2018). Maintenance of fish health in aquaculture: Review of epidemiological approaches for prevention and control of infectious disease of fish. Vet. Med. Int..

[8] Rodgers C.J., Furones M.D. (2009). Antimicrobial agents in aquaculture: Practice, needs and issues. Options Méditerr..

[9] Soltani M., Ghosh K., Hoseinifar S.H., Kumar V., Lymbery A.J., Roy S., Ringø E. (2019). Genus Bacillus, promising probiotics in aquaculture: Aquatic animal origin, bio-active components, bioremediation and efficacy in fish and shellfish. Rev. Fish Sci. Aquacult..

[10] Valipour A., Nedaei S., Noori A., Khanipour A.A., Hoseinifar S.H. (2019). Dietary Lactobacillus plantarum affected on some immune parameters, air-exposure stress response, intestinal microbiota, digestive enzyme activity and performance of narrow clawed crayfish (*Astacus leptodactylus*, Eschscholtz). Aquaculture.

[11] Hegde A., Kabra S., Basawa R.M., Khile D.A., Abbu R.U.F., Thomas N.A., Manickam N.B., Raval R. (2023). Bacterial diseases in marine fish species: Current trends and future prospects in disease management. World J. Microbiol. Biotechnol..

[12] Wang D., Liu H.B., Luo T.Y. (2016). Research progress on synergistic effect of Chinese herbal medicine and probiotics in aquaculture: A review. J. Dalian Fish. Univ..

[13] Li M., Wei D., Huang S., Huang L., Xu F., Yu Q., Liu M., Li P. (2022). Medicinal herbs and phytochemicals to combat pathogens in aquaculture. Aquacult. Int..

[14] Hai N.V. (2015). The use of probiotics in aquaculture. J. Appl. Microbiol..

[15] Nimrat S., Suksawat S., Boonthai T., Vuthiphandchai V. (2012). Potential Bacillus probiotics enhance bacterial numbers, water quality and growth during early development of white shrimp (*Litopenaeus vannamei*). Vet. Microbiol..

[16] Hosseini M., Miandare H.K., Hoseinifar S.H., Yarahmadi P. (2016). Dietary Lactobacillus acidophilus modulated skin mucus protein profile, immune and appetite genes expression in gold fish (*Carassius auratus gibelio*). Fish Shellfish Immunol..

[17] Kaur I.P., Kuhad A., Garg A., Chopra K. (2009). Probiotics: Delineation of prophylactic and therapeutic benefits. J. Med. Food.

[18] Taoka Y., Maeda H., Jo J.-Y., Jeon M.-J., Bai S.C., Lee W.-J., Yuge K., Koshio S. (2006). Growth, stress tolerance and non-specific immune response of Japanese flounder *Paralichthys olivaceus* to probiotics in a closed recirculating system. Fish. Sci..

[19] Biswas G., Korenaga H., Nagamine R., Takayama H., Kawahara S., Takeda S., Kikuchi Y., Dashnyam B., Kono T., Sakai M. (2013). Cytokine responses in the Japanese pufferfish (*Takifugu rubripes*) head kidney cells induced with heat-killed probiotics isolated from the Mongolian dairy products. Fish Shellfish Immunol..

[20] Chizhayeva A., Amangeldi A., Oleinikova Y., Alybaeva A., Sadanov A. (2022). Lactic acid bacteria as probiotics in sustainable development of aquaculture. Aquat. Living Res..

[21] Rohani F., Islam S.M., Hossain K., Ferdous Z., Siddik M.A., Nuruzzaman M., Padeniya U., Brown C., Shahjahan M. (2022). Probiotics, prebiotics and synbiotics improved the functionality of aquafeed: Upgrading growth, reproduction, immunity and disease resistance in fish. Fish Shellfish Immunol..

[22] Kim D., Beck B.R., Heo S.-B., Kim J., Kim H.D., Lee S.-M., Kim Y., Oh S.Y., Lee K., Do H. (2013). Lactococcus lactis BFE920 activates the innate immune system of olive flounder (*Paralichthys olivaceus*), resulting in protection against *Streptococcus iniae* infection and enhancing feed efficiency and weight gain in large-scale field studies. Fish Shellfish Immunol..

[23] Picchietti S., Fausto A.M., Randelli E., Carnevali O., Taddei A.R., Buonocore F., Scapigliati G., Abelli L. (2009). Early treatment with Lactobacillus delbrueckii strain induces an increase in intestinal T-cells and granulocytes and modulates immune-related genes of larval *Dicentrarchus labrax* (L.). Fish Shellfish Immunol..

[24] Zuo Z.-H., Shang B.-J., Shao Y.-C., Li W.-Y., Sun J.-S. (2018). Screening of intestinal probiotics and the effects of feeding probiotics on the growth, immune, digestive enzyme activity and intestinal flora of *Litopenaeus vannamei*. Fish Shellfish Immunol..

[25] Hoseinifar S.H., Hosseini M., Paknejad H., Safari R., Jafar A., Yousefi M., Doan H.V., Mozanzadeh M.T. (2019). Enhanced mucosal immune responses, immune related genes and growth performance in common carp (*Cyprinus carpio*) juveniles fed dietary *Pediococcus acidilactici* MA18/5M and raffinose. Dev. Comp. Immunol..

[26] Xia Y., Lu M., Chen G., Cao J., Gao F., Wang M., Liu Z., Zhang D., Zhu H., Yi M. (2018). Effects of dietary *Lactobacillus rhamnosus* JCM1136 and *Lactococcus lactis* subsp. *lactis* JCM5805 on the growth, intestinal microbiota, morphology, immune response and disease resistance of juvenile Nile tilapia, *Oreochromis niloticus*. Fish Shellfish Immunol..

[27] Beck B.R., Kim D., Jeon J., Lee S.-M., Kim H.K., Kim O.-J., Lee J.I., Suh B.S., Do H.K., Lee K.H. (2015). The effects of combined dietary probiotics *Lactococcus lactis* BFE920 and *Lactobacillus plantarum* FGL0001 on innate immunity and disease resistance in olive flounder (*Paralichthys olivaceus*). Fish Shellfish Immunol..

[28] Dawood M.A.O., Koshio S., Zaineldin A.I., Yokoyama S., Van Doan H., Moustafa E.M., Abdel-Daim M.M., Esteban M.A., Hassaan M.S. (2019). Dietary supplementation of selenium nanoparticles modulated systemic and mucosal immune status and stress resistance of red sea bream (*Pagrus major*). Fish Physiol. Biochem..

[29] Doan H.V., Hoseinifar S.H., Tapingkae W., Tongsiri S., Khamtavee P. (2016). Combined administration of low molecular weight sodium alginate boosted immunomodulatory, disease resistance and growth enhancing effects of *Lactobacillus plantarum* in Nile tilapia (*Oreochromis niloticus*). Fish Shellfish Immunol..

[30] Song Y., Zheng W., Zhang M., Cheng X., Cheng J., Wang W., Zhang J., Li Y. (2020). Out-of-season artificial reproduction techniques of cultured female tongue sole (*Cynoglossus semilaevis*): Broodstock management, administration methods of hormone therapy and artificial fertilization. Aquaculture.

[31] Guan C., Ding Y., Ma A., Wang Y., Li J., Ni Q., Liu X., Wang Q., Mai K., Lin H., Gui J., Tang Q., Li Z., Liu J., De Silva S.S. (2018). Flatfish farming. Aquaculture in China.

[32] Li Y., Yang Y., Song L., Wang J., Hu Y., Yang Q., Cheng P., Li J. (2021). Effects of dietary supplementation of Lactobacillus plantarum and Bacillus subtilis on growth performance, survival, immune response, antioxidant capacity and digestive enzyme activity in olive flounder (*Paralichthys olivaceus*). Aquac. Fish..

[33] Wei M., Xu W.-T., Gan T., Wang L., Zhang H.-X., Zhao F.-Z., Chen S.-L. (2019). Cloning, expression profile, and immune characterization of a novel stat5bl in Chinese tongue sole (*Cynoglossus semilaevis*). Fish Shellfish Immunol..

[34] Livak K.J., Schmittgen T.D. (2001). Analysis of relative gene expression data using real-time quantitative PCR and the 2^−ΔΔCt^ method. Methods.

[35] Yang K.M., Jiang Z.Y., Zheng C.T., Wang L., Yang X.F. (2014). Effect of Lactobacillus plantarum on diarrhea and intestinal barrier function of young piglets challenged with enterotoxigenic Escherichia coli K88. J. Anim. Sci..

[36] Mohammed E.A.H., Ahmed A.E.M., Kovács B., Pál K. (2025). The significance of probiotics in aquaculture: A review of research trend and latest scientific findings. Antibiotics.

[37] Dawood M.A., Koshio S., Ishikawa M., Yokoyama S., El Basuini M.F., Hossain S., Nhu T.H., Dossou S., Moss A.S. (2016). Effects of dietary supplementation of *Lactobacillus rhamnosus* or/and *Lactococcus lactis* on the growth, gut microbiota and immune responses of red sea bream (*Pagrus major*). Fish Shellfish Immunol..

[38] Kong Y., Li M., Chu G., Liu H., Shan X., Wang G., Han G. (2021). The positive effects of single or conjoint administration of lactic acid bacteria on *Channa argus*: Digestive enzyme activity, antioxidant capacity, intestinal microbiota and morphology. Aquaculture.

[39] Munir M.B., Hashim R., Chai Y.H., Marsh T.L., Nor S.A.M. (2016). Dietary prebiotics and probiotics influence growth performance, nutrient digestibility and the expression of immune regulatory genes in snakehead (*Channa striata*) fingerlings. Aquaculture.

[40] Van Doan H., Lumsangkul C., Jaturasitha S., Meidong R., Hoseinifar S.H., Dawood M.A. (2021). Modulation of growth, skin mucus and serum immunities, and disease resistance of Nile tilapia fed host-associated probiotic (*Lactobacillus paracasei* L61-27b). Aquacult. Nutr..

[41] Sun Y.Z., Yang H.L., Ma R.L., Song K., Li J.S. (2012). Effect of *Lactococcus lactis* and *Enterococcus faecium* on growth performance, digestive enzymes and immune response of grouper *Epinephelus coioides*. Aquacult. Nutr..

[42] He G., Sun H., Liao R., Wei Y., Zhang T., Chen Y., Lin S. (2022). Effects of herbal extracts (*Foeniculum vulgare* and *Artemisia annua*) on growth, liver antioxidant capacity, intestinal morphology and microorganism of juvenile largemouth bass (*Micropterus salmoides*). Aquacult. Rep..

[43] Hellou J., Ross N.W., Moon T.W. (2012). Glutathione, glutathione S-transferase, and glutathione conjugates, complementary markers of oxidative stress in aquatic biota. Environ. Sci. Pollut. Res..

[44] Shah S.S., Stone E.F., Francis R.O., Karafin M.S. (2024). The global role of G6PD in infection and immunity. Front. Immunol..

[45] Jiang D.X., Liu W.H., Zhang C.N., Zhou Y. (2025). Application of lactic acid bacteria in the growth of aquatic animals. Anim. Breed. Feed.

[46] Kumari A., Pathak D.P., Asthana S. (2020). Bile acids mediated potential functional interaction between FXR and FATP5 in the regulation of lipid metabolism. Int. J. Biol. Sci..

[47] Kingkaew E., Konno H., Hosaka Y., Phongsopitanun W., Tanasupawat S. (2023). Characterization of lactic acid bacteria from fermented fish (pla-paeng-daeng) and their cholesterol-lowering and immunomodulatory effects. Microbes Environ..

[48] Chrostek L., Supronowicz L., Panasiuk A., Cylwik B., Gruszewska E., Flisiak R. (2013). The effect of the severity of liver cirrhosis on the level of lipids and lipoproteins. Clin. Exp. Med..

[49] Helkin A., Stein J.J., Lin S., Siddiqui S., Maier K.G., Gahtan V. (2016). Dyslipidemia Part 1—Review of lipid metabolism and vascular cell physiology. Vasc. Endovasc. Surg..

[50] Dawood M.A., Abo-Al-Ela H.G., Hasan M.T. (2020). Modulation of transcriptomic profile in aquatic animals: Probiotics, prebiotics and synbiotics scenarios. Fish Shellfish Immunol..

[51] Ichiki S., Kato-Unoki Y., Somamoto T., Nakao M. (2012). The binding spectra of carp C3 isotypes against natural targets independent of the binding specificity of their thioester. Dev. Comp. Immunol..

[52] Zhao J., Zhao Y., Liu H., Cao Q., Feng L., Zhang Z., Jiang W., Wu P., Liu Y., Luo W. (2023). Dietary leucine improves fish intestinal barrier function by increasing humoral immunity, antioxidant capacity, and tight junction. Int. J. Mol. Sci..

[53] Paritova A., Nurgaliyev A., Nurgaliyeva G., Abekeshev N., Abuova A., Zakirova F., Zwierzchowski G., Kuanchaleyev Z., Issabekova S., Kizatova M. (2024). The dietary effects of two strain probiotics (*Leuconostoc mesenteroides*, *Lactococcus lactis*) on growth performance, immune response and gut microbiota in Nile tilapia (*Oreochromis niloticus*). PLoS ONE.

[54] Li R., Chi T., Xu Q., Liu J., Shan X., Zhou R., Yao J., Sun W., Wang G. (2023). Effects of single or conjoint administration of lactic acid bacteria as potential probiotics on the growth, immune responses, and disease resistance of *Carassius auratus*. Aquacult. Int..

[55] Atiba A., Nishimura M., Kakinuma S., Hiraoka T., Goryo M., Shimada Y., Ueno H., Uzuka Y. (2011). Aloe vera oral administration accelerates acute radiation-delayed wound healing by stimulating transforming growth factor-β and fibroblast growth factor production. Am. J. Surg..

[56] Deng Z., Fan T., Ao C., Tian H., Zheng Y., Li C., He J. (2024). TGF-β signaling in health, disease and therapeutics. Signal Transduct. Target Ther..

[57] Tian Q., Yang Z., Zang T., Li Q., Chen W., Meng L., Wang A. (2024). Effects of lactic acid bacteria on growth, antioxidant capacity, and intestinal health of *Micropterus salmoides*. Feed Ind..

